# Phthalate Esters and Their Potential Risk in PET Bottled Water Stored under Common Conditions

**DOI:** 10.3390/ijerph17010141

**Published:** 2019-12-24

**Authors:** Xiangqin Xu, Gang Zhou, Kun Lei, Gerald A. LeBlanc, Lihui An

**Affiliations:** 1 State Key Laboratory of Environmental Criteria and Risk Assessment, Chinese Research Academy of Environmental Sciences, Beijing 100012, China; 2 Department of Biological Sciences, North Carolina State University, Raleigh, NC 27606, USA

**Keywords:** PET bottles, contained water, PAEs, migration, health risk

## Abstract

A great deal of attention has been paid lately to release of phthalate esters (PAEs) from polyethylene terephthalate (PET) bottles into PET bottled drinking water due to their potential endocrine-disrupting effects. Three kinds of PAEs, including diethyl phthalate (DEP), dimethyl phthalate (DMP) and dibutyl phthalate (DBP), were detected in 10 popular brands of PET bottles in Beijing, ranging from 101.97 μg/kg to 709.87 μg/kg. Meanwhile, six kinds of PAEs, including DEP, DMP, DBP, n-butyl benzyl phthalate (BBP), di-n-octyl phthalate (DOP) and di(2-ethylhexyl) phthalate (DEHP), were detected in PET bottled water, ranging from 0.19 μg/L to 0.98 μg/L, under an outdoor storage condition, while their concentrations ranged from 0.18 μg/L to 0.71 μg/L under an indoor storage condition. Furthermore, the concentrations of PAEs in brand D and E bottles were slightly increased when the storage time was prolonged. In addition, the concentrations of PAEs in commercial water contained in brand B and H bottles and pure water contained in brand E and G bottles were also slightly increased with the increase of storage temperature. Interestingly, DBP mainly contributed to the increased PAEs levels in simulation water. These results suggest that a part of the PAEs in PET bottled water originated from plastic bottles, which was related to the storage time and temperature. However, the PAEs in PET bottled water only pose a negligible risk to consumers if they follow the recommendations, such as storage at a common place (24 °C), away from sun and in a short period of time.

## 1. Introduction

Drinking water stored in polyethylene terephthalate (PET) bottles has become popular due to convenience and low cost. Recently, Transparency Market Research has estimated that the global market of bottled water would grow rapidly (6.44% per annum) between 2017 and 2024 [1]. However, public health concerns related to the potential leaching of chemicals such as phthalate esters (PAEs) from PET bottles into the drinking water have been raised [2,3] due to their potential endocrine-disrupting effects [4].

PAEs are a group of chemicals used as plasticizers in plastic products to improve softness and flexibility [5]. However, PAEs can leach from thermoplastic packages into foods, beverages, and drinking water since they are not part of the polymeric matrix. For example, three kinds of PAEs, dibutyl phthalate (DBP), diethyl phthalate (DEP) and di(2-ethylhexyl) phthalate (DEHP), have been detected in PET bottled water [6]. Moreover, the concentrations of DBP, DEP and DEHP in PET bottled water are nearly 20-fold higher compared with water contained in glass bottles [6,7]. Leaching of PAEs from PET bottles is affected by storage conditions, mainly including temperature and time. For example, a case study reported that storage of water in PET bottles for 10 weeks at an outdoor temperature up to 30 °C results in increased concentrations of n-butyl benzyl phthalate (BBP), DBP and DEHP in drinking water [7]. A recent study demonstrated that there was no significant leaching of DBP, BBP or DEHP from PET bottles to drinking water when stored at low temperatures [8]. In addition, the concentrations of PAEs in soft beverages stored in PET bottles (pH = 3) were measured as between 5- and 40-fold higher compared with mineral water (pH = 5) [9], suggesting that pH might affect the leaching of PAEs from PET bottles. However, no difference was observed between water and carbonated water stored in PET bottles [6]. Contamination of PAEs in water stored in PET bottles had been observed, which was hypothesized to be attributable to (1) water contamination in the bottling plant, (2) migration of PAEs from bottle material to water and (3) cross-contamination during analytical procedure [3]. Therefore, it is necessary to unveil the sources of PAEs in PET bottled drinking water and evaluate the potential health risk due to the increasing consumption of PET bottled drinking water globally.

To understand the leaching characteristics of PAEs from PET bottles into water under normal storage scenarios, the present study examined the concentrations of PAEs in PET bottles and PET bottled water. Moreover, the leaching characteristics of PAEs from PET bottles into water stored under typical conditions were evaluated. Lastly, we also assessed the health risk associated with PAEs in the water.

## 2. Material and Methods

### 2.1. Consumer Survey and Brand Selection

Typical-use information related to bottled water was collected by direct interviews from 204 consumers who bought bottled water at 17 supermarkets in Beijing. Consumers were asked questions face to face, about topics such as their favorite brand, frequency of bottled water consumption and storage conditions. For most consumers (>70%), the intermediate-sized PET bottled water was preferred. Bottles of PET bottled water from the 10 most popular brands (≈500 mL) with similar production dates were purchased in the summer of 2016 from Wumart supermarket in Beijing. Brands of bottled water were labeled with a letter from A to J (Table 1).

### 2.2. Chemical Reagents

The compounds of BBP, DBP, DEHP, DEP, dimethyl phthalate (DMP) and di-*n*-octyl phthalate (DOP) with a purity of 98% were purchased from Manhage Bio-Tech (Beijing, China). Other solvents and chemicals were of analytical or HPLC grade.

### 2.3. Measurement of PAEs in PET Bottles

Purchased PET bottles were emptied, rinsed with Milli-Q water and dried. Bottles were cut into small pieces of less than 0.2 mm in size using a pair of scissors, followed by analysis of PAEs contents according to the National Standard Methods for the Analysis of PAEs (GB/T21928-2008, China). Briefly, a 0.3 g plastic piece was treated with 20 mL of hexane (Merck, Darmstadt, Germany) under ultrasonic extraction for 30 min. After filtration, this process was repeated on residual plastic two more times. The three batches of 20 mL aliquots were pooled, transferred to a glass bottle, and dried by rotary evaporation at 45 °C. The dried sample was dissolved in 0.5 mL of hexane for PAE analysis. Three replicates were conducted for each brand of PET bottle.

### 2.4. Sample Treatment

#### 2.4.1. Storage of PET Bottled Water under Normal Conditions

The consumer survey demonstrated that more than 80% of consumers frequently stored bottled water in a car trunk for up to 4 weeks. Therefore, PET bottled drinking water was stored in a car trunk for over 4 weeks in July. Meanwhile, PET bottled drinking water of the same brands was stored at room temperature (24 ± 1 °C) in the dark. Bottled water was sampled after 1, 2 and 4 weeks of storage for PAE analysis.

#### 2.4.2. Effect of Temperature on the Concentration of PAEs in Commercial Bottled Water

Bottled water of the same brands as mentioned above was stored in incubators at 40 °C, 50 °C, 60 °C and 70 °C, respectively. After 24 h, bottled water was sampled at the above-mentioned time points for PAE analysis (*n* = 3).

#### 2.4.3. Effect of Storage Temperature on the Concentrations of PAEs in Pure Water

To further evaluate the potential leaching of PAEs from PET bottles, Millipore water made in a lab (Milli-Q Advantage, Germany) was poured into the empty PET bottles of the same brands as mentioned above, followed by incubation at 40 °C, 50 °C, 60 °C and 70 °C for 24 h. Water samples (*n* = 3) were treated with the same procedure for PAE analysis.

### 2.5. Water Preparation

Water samples were prepared for analysis of PAEs according to Li et al. [10]. Bottled water (≈1.0 L) was passed through the preconditioned C18 column, and then the column was completely dried under nitrogen. The target chemicals were eluted and concentrated into 0.5 mL hexane for subsequent analysis.

### 2.6. PAEs Analysis

Sample extracts were analyzed by gas chromatography coupled with mass spectrometry (Shimadzu, Tokyo, Japan) according to Li et al. [10]. The ion source temperature was set at 230 °C, and the interface temperature was maintained at 280 °C after a series of temperature-increasing processes. To monitor the target chemicals, the multiple reaction monitoring (MRM) mode was used to identify mass spectra, and a 2.0 µL sample was injected in splitless mode at an inlet temperature of 280 °C. The instrumental limit of detection (LOD) and limit of quantification (LOQ) for the six target PAEs ranged from 0.035 to 0.28 ng/L and from 0.11 to 0.66 ng/L, respectively. Recovery was 97.50% ± 8.86%, and precision was 3.62% ± 1.99%. The standard curve was established with a mixture of six kinds of PAEs, and the abundances with strong linearity (*r^2^* > 0.999) were obtained.

All tools, including beakers and funnels, were washed three times with acetone, rinsed three times with dichloromethane and dried at 350 °C to minimize contamination and maximize accuracy. Each batch of 10 samples was analyzed along with two procedural blanks, and results were corrected by subtracting blank values for DBP and DEHP, which were detected in procedural blanks.

### 2.7. Risk Assessment

Health risk of the PAEs was estimated using the Hazard Quotient (*HQ*) method (USEPA, https://www.chemsafetypro.com/Topics/CRA/How to Calculate Hazard Quotients (*HQ*) and Risk Quotients (RQ).html). The *HQ* is defined as the ratio of the potential exposure to a substance and the level at which no adverse effects are expected. Typically, the *HQ* is calculated as follows:

$$HQ=\frac{EDI}{RfD}$$

where *EDI* is the expected daily intake, and *RfD* is the reference dosage. *RfD* is the product of the no adverse effect level, as established from epidemiological studies or toxicity evaluations in animal models and appropriate uncertainty factors. In the present study, the *EDI* was calculated as the concentration (μg/L) of the PAE divided by an average adult weight of 60 kg divided by 1 L (two bottles of water per day per person). *RfDs* for the individual PAEs were obtained from the IRIS database (USEPA, https://www.epa.gov/iris). *RfDs* were available only for DEHP, DEP, DBP and BBP. *HQ* of >1 is indicative of significant risk, whereas *HQ* of <1 is indicative of low risk.

### 2.8. Data Analysis

Data were expressed as mean ± standard deviation (SD), and SD was consistently less than 20% of the mean value. Significant differences in PAE concentrations among samples were analyzed using one-way analysis of variance, followed by Tukey’s post-hoc test (SPSS software Ver.13.0, SPSS Inc., Chicago, IL, USA). Significant difference was considered at *p* < 0.05.

## 3. Results

### 3.1. Concentrations of PAEs in PET Bottles

**Figure 1:**
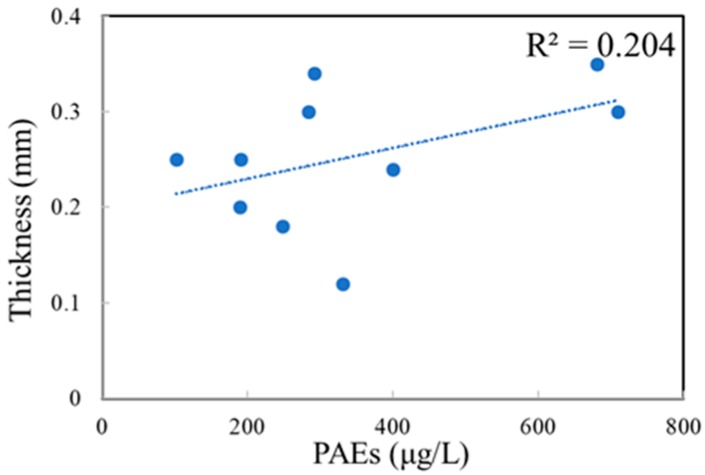
Correlations between the bottle thickness and amount of phthalate esters (PAEs) in polyethylene terephthalate (PET) bottles.

Three kinds of PAEs, including DEP, DMP and DBP, were detected in all 10 brands of PET bottles (Table 2), and there was weak positive correlation between the bottle thickness and amount of PAEs in PET bottles (Figure 1). The total concentration of the PAEs ranged from 101.97 µg/kg in brand J to 709.87 μg/kg in brand I. DBP was the most abundant compound, accounting for over 40% of PAEs in all brands, and its content was as much as 83.62% in brand D. The concentration of DBP was the greatest in brand I bottles (511.52 μg/kg), while its lowest value was detected in brand J bottles (62.90 μg/kg). Interestingly, BBP, DOP and DEHP were not detected in any brands of the PET bottles. These results were different from those for plastic food containers, in which only DEHP was detected at concentrations ranging from 83 to 127 μg/kg [11].

### 3.2. Concentrations of PAEs in Commercial Bottled Water Stored under Common Conditions

**Figure 2:**
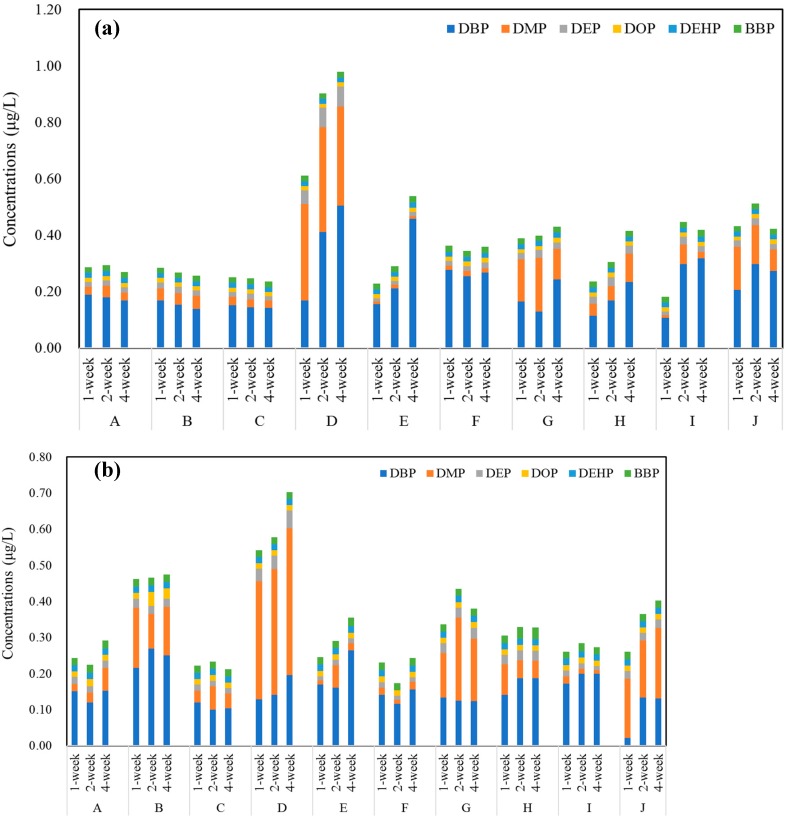
Total concentrations (μg/L) of PAEs in each brand of PET bottled water stored (**a**) outdoors and (**b**) in lab for 4 weeks, respectively (*n* = 3).

All six kinds of PAEs were detected in the 10 different brands of commercial bottled water stored under outdoor conditions (Figure 2a). The total concentration of PAEs ranged from 0.19 to 0.98 μg/L with an average value of 0.39 μg/L. Similarly, the concentrations of PAEs in PET bottled water stored under indoor conditions ranged from 0.18 to 0.71 μg/L, with an average value of 0.34 μg/L (Figure 2b). Among the 10 brands, the highest concentrations of PAEs were detected in brand D, no matter if they were stored outdoors or indoors. No significant differences were observed between concentrations of PAEs in water stored indoors or outdoors (*p* > 0.05). However, the total concentration of PAEs in brands D and E showed a weak increasing trend, which was mainly attributed to the increase of DBP concentration. Among these compounds, DBP and DMP were the most common and abundant PAEs, accounting for more than 60% of total PAEs measured in all samples. Compared with the available data, the concentration of DEHP was lower than WHO standards of 8 μg/L [12], China standards of 8 μg/L [13] and US EPA standards of 6 μg/L [14].

### 3.3. Concentrations of PAEs in Commercial Bottled Water Stored at Different Temperatures

**Figure 3:**
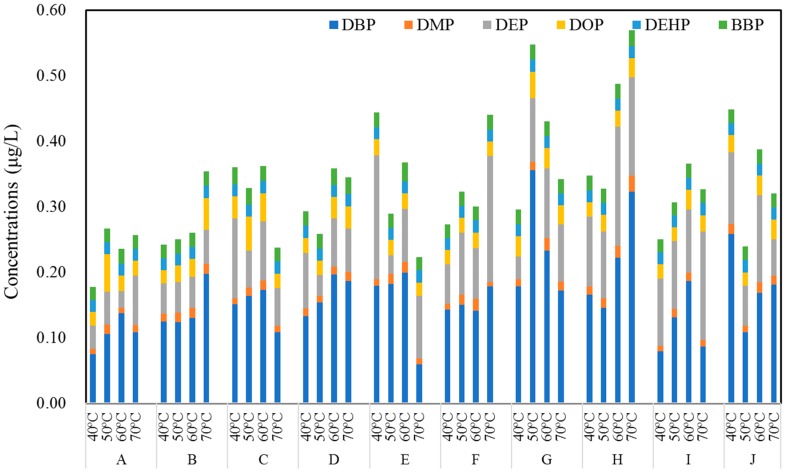
Total concentrations (μg/L) of PAEs in each brand of PET bottled water from a local supermarket after incubation at 40, 50, 60 and 70 °C for 24 h (*n* = 3).

**Figure 4:**
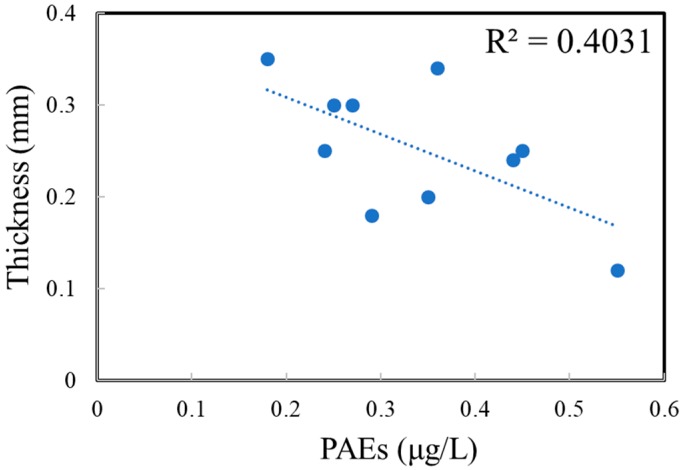
Correlations between bottle thickness and amount of PAEs stored at 40 °C.

The total concentrations of PAEs in commercial bottled water stored in PET bottles at 40, 50, 60 and 70 °C were 0.31 ± 0.09, 0.31 ± 0.09, 0.36 ± 0.07 and 0.34 ± 0.10 μg/L, respectively (Figure 3). Moreover, there was a weak negative correlation between the bottle thickness and the amount of PAEs in commercial water (Figure 4). However, the PAE concentrations remained unchanged with the increase of temperature, and the PAE concentrations were independent of water types. No significant difference was observed among compounds in water stored in PET bottles from the 10 brands, except for brand B and H, which also showed a weak increasing trend (*p* > 0.05). Similarly, the concentrations of DBP, DMP and DEP accounted for more than 60% of total PAEs in all samples, of which the DBP was the most abundant compound, and the concentration of DEHP was also lower than the available standards of WHO (8 μg/L), China (8 μg/L), and USEPA (6 μg/L) [12,13,14].

### 3.4. Concentrations of PAEs in Pure Water Stored at Different Temperatures

**Figure 5:**
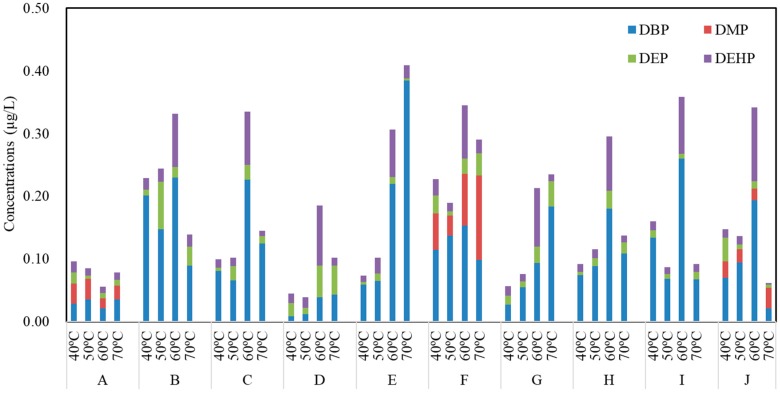
Total concentrations (μg/L) of PAEs in pure water stored in 10 brands of PET bottles after incubation at 40, 50, 60 and 70 °C for 24 h (*n* = 3).

Three kinds of PAEs were detected in pure water samples, including DBP (0.011 μg/L), DEP (0.001 μg/L) and DOP (0.005 μg/L), implying that contamination by PAEs was common in the present environment besides lab conditions. Moreover, four kinds of PAEs were detected in pure water samples collected from PET bottles (Figure 5), whereas six PAEs were detected from commercial water collected from PET bottles, which were different from those detected in the pure water. Namely, DMP (0.02–0.13 μg/L) was only detected in renewed pure water from the brands A, F and J bottles, whereas three compounds were detected in all other brands, ranging from 0.005 to 0.08 μg/L for DEP, from 0.01 to 0.38 μg/L for DBP, and from 0.01 to 0.09 μg/L for DEHP, which were also lower than the standards of WHO (8 μg/L), China (8 μg/L) and USEPA (6 μg/L) [12,13,14]. The total concentration of PAEs ranged from 0.04 to 0.41 μg/L, and no positive relationship was found between the PAEs concentrations and storage temperature. However, the PAE concentration was slightly increased in brand E and G with the increase of temperature (*p* > 0.05), and this increase could be mainly attributed to DBP. This finding was also similar to the result of brand B and H bottles containing commercial water.

### 3.5. Health Risk Assessment

Risk of adverse health outcome was evaluated based on four kinds of PAEs (DEP, DBP, BBP and DEHP) in PET bottled water, for which RfDs were available. HQs for these PAEs associated with the consumption of water stored in PET bottles were far less than 1 (Table 3). As expected, most consumers would face negligible health risks from these four PAEs in PET bottled water if drinking two bottles of water daily stored under normal conditions. 

## 4. Discussion

Recently, leaching of DBP, DEP and DEHP from thermoplastic products has been studied due to their potential to affect the endocrine system [3,15]. Previous evidence has demonstrated that DBP, DEP and DEHP can be released from PET bottles into water when stored under various conditions (such as long times, high temperature, and sunlight). Similarly, our data also confirmed that DBP was released into water from PET bottles when the storage time was prolonged and the storage temperature was increased, although no positive relationship was found between the concentrations of PAEs and storage conditions. Moreover, DBP was the main contributor to the increase of PAEs concentrations. These results were consistent with the data of Diana and Dimitra [3] and Keresztes et al. [16], implying that temperature was not the only factor influencing the concentrations of PAEs in water stored in PET bottles. All of these findings indicate that there might be other sources of PAEs in water stored in PET bottles besides the bottles themselves.

Diana and Dimitra [3] speculated that PAEs in bottled water may be derived from numerous pathways as follows: (1) leaching from poor-quality bottle materials, (2) source contamination during bottling in plants from usage of plastic pipes and (3) experimental contamination during the analytical procedure due to the wide use of plastic tools. In the present study, we investigated the source of PAEs in water samples by first evaluating PAEs in the plastic material of PET bottles as well as in the water contained in these bottles. Two or three kinds of PAEs were detected in the 10 PET bottles, while six kinds of PAEs were detected in water stored in bottles of the same brands. These findings suggested that the PET bottles were not the sole source of PAEs in the bottled water. Therefore, the water might be contaminated with PAEs prior to bottling. PAEs have been detected in tap water [17] and were even detected in pure water in the current study, indicating that PAEs were ubiquitous environmental pollutants, and they could be found in various environments [18,19,20]. Therefore, more strict measurements should be taken to eliminate the potential risk of chemical compounds in PET bottled drinking water during the whole production process [21].

In addition, three kinds of PAEs were detected in laboratory pure water, the concentrations of these PAEs were increased with the storage time, and additional PAEs were also detected. These results suggest that some PAEs were transferred from PET bottles to water. However, leaching appeared to be independent of storage temperature over a short period of time. Therefore, PAEs detected in water stored in PET bottles were more likely to be originated, at least in part, from the bottles themselves. Importantly, the concentrations of DEHP were much lower compared with the standards of USEPA (6.0 μg/L) and WHO (8.0 μg/L), even when stored at 70 ºC for 24 h. In contrast, Ceretti et al. [22] found that no trace of DEHP or DBP was detected in water stored in PET bottles, even when stored at 40 °C for 10 days. Such discrepancy might be attributed to differences in composition of bottles between studies [3,6]. Another reason might be related to ultraviolet (UV) light. A recent study showed that poor storage conditions increase the PAE (DEP, DBP and DEHP) concentrations in PET bottles, which can be possibly attributed to increased exposure to UV radiation from sunlight [7]. In addition, UV radiation increases the leaching of various chemicals from plastic bottles and other materials, such as additives and PAEs [23,24]. In the present study, both indoor and outdoor simulation tests were performed without light, which might retard the release of PAEs. So, the recommended conditions, such as storage at a common place (24 °C), away from sun and in a short time, should be followed by consumers to preserve the quality of bottled water.

Furthermore, we evaluated the risks associated with only four PAEs individually. The results showed that no matter the source of PAEs in PET bottled water, the health risk associated with the levels of selected PAEs was acceptable for adults, which was consistent with a previous report [8]. However, caution should be exercised in interpreting this assessment, as humans are exposed to PAEs through multiple routes in addition to bottled drinking water. This means that humans can be exposed to additional PAEs, and some of these compounds may additively act to elicit toxicity. Therefore, it is necessary to assess the potential health risks of PAEs in future work as the consumption of plastic-bottled water is continuously increasing [23].

## 5. Conclusions

PAEs, including DEP, DMP and DBP, were detected in PET bottles, while additional compounds with three kinds of PAEs were detected in water contained in PET bottles stored under common storage scenarios (such as storage time and temperature). The investigation demonstrated that the plastic bottles were not the sole source of PAEs, and more strict measurements should be taken to reduce the potential health risk of PAEs in PET bottled drinking water. Importantly, the release of PAEs from PET bottles to water could be retarded by avoiding high temperatures, long storage time and UV radiation during storage. As expected, the concentrations of PAEs in PET bottled drinking water were negligible under common storage conditions for consumers. Of course, further research is necessary to assess the potential risk for human health owing to direct and indirect human exposure via different pathways, including water, food and air.

## Figures and Tables

**Table 1:** Commercial Bottled Water Evaluated in the Study.

Brands	Product Date	Bottle Size	Bottle Color	Bottle Thickness/mm	Bottled Water	Price (¥/bottle)
**A**	March, 2016	510 mL	Clear	0.35	Natural mineral water	5.10
**B**	March, 2016	570 mL	Light blue	0.25	Natural mineral water	2.30
**C**	February, 2016	600 mL	Clear	0.34	Natural mineral water	2.80
**D**	March, 2016	550 mL	Light blue	0.18	Purified drinking water	2.70
**E**	February, 2016	550 mL	Clear	0.24	Natural mineral water	1.10
**F**	February, 2016	500 mL	Clear	0.30	Natural mineral water	2.30
**G**	March, 2016	550 mL	Light blue	0.12	Purified drinking water	2.10
**H**	March, 2016	596 mL	Clear	0.20	Purified drinking water	1.40
**I**	March, 2016	555 mL	Clear	0.30	Purified drinking water	1.70
**J**	March, 2016	550 mL	Light blue	0.25	Purified drinking water	1.40

**Table 2:** Concentrations (μg/kg) of PAEs in PET bottles.

	A	B	C	D	E	F	G	H	I	J
**DEP**	182.43 ± 5.03	29.92 ± 2.11	50.73 ± 4.12	49.47 ± 1.02	114.83 ± 4.23	22.88 ± 1.44	45.78 ± 3.02	34.83 ± 2.35	68.08 ± 5.66	ND
**DMP**	188.58 ± 8.20	34.12 ± 3.35	41.90 ± 2.15	16.12 ± 0.44	9.07 ± 0.24	58.10 ± 8.41	133.93 ± 9.54	19.50 ± 2.01	130.27 ± 11.30	39.07 ± 5.20
**DBP**	310.12 ± 15.22	126.20 ± 7.84	199.52 ± 9.55	334.82 ± 6.45	159.32 ± 14.55	167.20 ± 7.74	150.57 ± 11.45	134.42 ± 15.44	511.52 ± 22.07	62.90 ± 8.04
**Total**	**681.13**	**190.23**	**292.15**	**400.40**	**283.22**	**248.18**	**330.28**	**188.75**	**709.87**	**101.97**

n-butyl benzyl phthalate (BBP), di-n-octyl phthalate (DOP) and di(2-ethylhexyl) phthalate (DEHP) were not detected. ND: not detected.

**Table 3:** Health risk assessment of PAEs (µg/L) for adults via consumption of PET bottled waters stored under typical conditions.

	Storage at Indoor (24 ± 1 °C)				Storage at Outdoor (22.5–44.4 °C)			
	DEHP	DEP	DBP	BBP	DEHP	DEP	DBP	BBP
Maximum Concentration	0.02	0.05	0.26	0.03	0.02	0.07	0.51	0.03
EDI ^a^	0.0003	0.0008	0.0043	0.0005	0.0003	0.0012	0.0085	0.0005
RfD ^b^	20	800	100	200	20	800	100	200
HQ ^c^	1.67 × 10^−5^	1.04 × 10^−6^	4.33 × 10^−5^	2.50 × 10^−6^	1.67 × 10^−5^	1.46 × 10^−6^	8.50 × 10^−5^	2.50 × 10^−6^
	**Storage at 40 °C**				**Storage at 70 °C**			
	**DEHP**	**DEP**	**DEHP**	**DEP**	**DEHP**	**DEP**	**DEHP**	**DEP**
Maximum Concentration	0.02	0.19	0.02	0.19	0.02	0.19	0.02	0.19
EDI ^a^	0.0003	0.0032	0.0003	0.0032	0.0003	0.0032	0.0003	0.0032
RfD ^b^	20	800	20	800	20	800	20	800
HQ ^c^	1.67 × 10^−5^	3.96 × 10^−6^	1.67 × 10^−5^	3.96 × 10^−6^	1.67 × 10^−5^	3.96 × 10^−6^	1.67 × 10^−5^	3.96 × 10^−6^

^a^ Estimated Daily Intake (EDI) via bottled drinking water (μg/Kg body weight/day). ^b^ IRIS RfD: chronic non-carcinogenic Reference Dose (RfD) (μg/Kg bw/day). ^c^ Hazard Quotient (EDI/RfD).
